# Green synthesis of silver nanoparticles using *Adhatoda vasica* leaf extract and its application in photocatalytic degradation of dyes

**DOI:** 10.1186/s11671-023-03914-5

**Published:** 2023-10-30

**Authors:** Ronak Kumar Chaudhari, Priyanka A. Shah, Pranav S. Shrivastav

**Affiliations:** 1 Department of Chemistry, School of Sciences, Gujarat University, Navrangpura, Ahmedabad, Gujarat 380009 India; 2 Department of Forensic Sciences, National Forensic Sciences University, Dharwad, Karnataka 580011 India

**Keywords:** Silver nanoparticles, Green bio synthesis, *Adhatoda vasica* leaf extract, Characterization, Dye degradation

## Abstract

The paper describes biogenic synthesis of silver nanoparticles (AgNPs) using *Adhatoda vasica* leaf extracts at room temperature. The prepared AgNPs were characterized by UV–visible spectroscopy, Fourier-transform infrared spectroscopy, powder X-ray diffraction, Energy dispersive X-ray (EDX), High Resolution Transmission Electron Microscope, Scanning Electron Microscopy and Thermogravimetric analyser. The bio reduction method is devoid of any toxic chemicals, organic solvents, and external reducing, capping and stabilizing agent. The synthesized AgNPs had spherical shape with particle size ranging between 3.88 and 23.97 nm and had face centered cubic structure. UV–visible spectral analysis confirmed the formation of AgNPs with a characteristic surface plasmon resonance band at 419 nm. The EDX pattern revealed the presence of elemental Ag in AgNPs. The prepared AgNPs were used for degradation of Amaranth, Allura red and Fast green in aqueous medium, with ≥ 92.6% efficiency within 15 min using 5 mg of AgNPs. The optical bandgap, Eg value of 2.26 eV for AgNPs was found to be effective for rapid photocatalytic degradation of all the three dyes. The degradation process was observed to follow pseudo first order kinetics.

## Introduction

Dyes industries are one such industrial sector that consumes large amounts of water and are responsible for the discharge of untreated effluents containing non-biodegradable dyes. Of approximately 1.0 million tons of dye produced annually, nearly 15% is lost in effluents from dyeing houses, causing serious environmental concerns due to their toxicity, carcinogenicity, and mutagenicity [1]. They are responsible for reduction in dissolved oxygen content, decrease in sunlight penetration in water bodies and formation of toxic substances on account of chemical or biological degradation pathways [2, 3]. Several approaches have been considered for the degradation of dyes, which includes physical, chemical, biological and radiation treatment. All these methodologies have their own advantages and limitations with several factors affecting their efficiency [4]. Moreover, some of these approaches generate secondary pollutants and thus there is a need to have alternative approaches that are more effective and economical to counter this perennial concern [4–6].

In the past decades, there has been a growing recognition of the potential of nanoparticles (NPs) in removing dyes from wastewater. This is due to their large surface area, strong adsorption capabilities, efficient diffusion, and quick attainment of equilibrium [7]. Although there are different physico-chemical methods like electro-spraying, laser pyrolysis, laser ablation and evaporation–condensation, combustion, thermal decomposition, sol–gel method, hydrothermal, ultrasonication, microwave-assisted combustion, and chemical reduction to prepare NPs [8–10], however, they require expensive experimental setup, consume higher energy, some involve use of toxic and hazardous chemicals, which are detrimental to the environment. Thus, green synthesis is widely applied to get large-scale yield of NPs, through cost effective and eco-friendly approach [11]. The biological entities like algae, bacteria, fungi and plant materials having an excellent reducing capacity, as they reduce the metal ion in zero valent state [12]. Different parts of plants such as fruit, flower, leaf, bark, seed and roots are very useful in green synthesis, as they have phytochemicals like flavonoids, phenols, terpenoids, sugar, ketones, aldehyde, carboxylic acid and amines that act as reducing as well as stabilizing agent [13].

Many metal-based NPs have been synthesised via green approach, but AgNPs have attracted significant attention as they possess excellent physical, chemical, and biological properties, making them suitable for a wide range of applications. This includes different fields like pharmaceuticals, electronics, biomedicine, drug delivery, biomedical devices, delivery of peptides, environmental monitoring, dye degradation, and in control of infectious diseases [7, 14, 15]. They also show anticancer, antifungal, antibacterial, antiprotozoal, and antimicrobial activity against gram-positive and gram-negative bacteria [13]. Raveendran and co-workers [16] were the first to report the synthesis of AgNPs using a green method. The process was simple and eco-friendly, wherein the solution of silver nitrate and β-d glucose was heated with an aqueous solution of starch to obtain monodispersed AgNPs. In their method, β-d glucose acted as a reducing agent while starch was used as a stabilizing agent. Since then, many researchers have prepared AgNPs using different plant materials like, *Diospyros lotus* (leaf) [17], *Berberis vulgaris* (leaf and root) [18], *Thymus kotschyanus* [19], *Sapota* (Fruit) [20], *Tulsi* (leaf) [21], *Zanthoxylum armatum* (leaf) [3], *Mussaendraerythrophylla* (leaf) [22], *Artemisia annua* (leaf) [23], *Saraca indica* (flower) [24], *Trapa bispinosa* (peel) [25], *Garlic* [26], *Vitex negundo* (Leaf) [27], *Medicago sativa* (seed) [28], *Nicotiana tobaccum* (leaf) [29], *Sorghum bicolor* (bran) [30], *Allium cepa* [31], *Azadirachta indica* (leaf) [32], *Gliricidiasepium* (leaf) [33], *Euphorbia hirta* (leaf) [34], *Boswellia Ovalifoliolata* (bark) [35], *Jatropha curcas* (latex) [36], *Carica papaya* (fruit) [37], *Cinnamomum camphora* (leaf) [38], *Capsicum annuum* [39], *Emblica officinalis* (fruit) [40], *Aloevera* [41], *Pelargonium graveolens* (leaf) [42], *Dalbergia sisoo* (leaf) [43] and *Angelica gigas* ribbed stem extracts [44]. Additionally, few other methods have reported the use of *Annona muricata L.* and *Acmella uliginosa* leaf extract to prepare efficient catalysts for photocatalytic degradation of malachite green dye [45, 46].

According to Ayurveda and Unani medicine, *Adhatoda vasica* is a very useful medicinal plant, and is used as an indigenous medicine for many centuries [47, 48]. It belongs to *Acanthaceae* family and contains alkaloids, triterpenoid, flavonoid, chalcone, steroid, alkyl ketones, alkyl hydroxyl ketone, glucoside, amino acid, and organic acids. These compounds are responsible for reduction and stabilization of metal ions [47]. Bhumi and co-workers synthesized AgNPs from *Adhatoda vasica* leaf extract and demonstrated their antifungal and antibacterial activity against *S. aureus, Pseudomonas aeuroginosa, E. coli, B. thuringiensis* and *K. Pneumonia* [49]. Catalytic degradation of methylene blue and methylene green dyes with AgNPs prepared from *Adhatoda vasica* leaf extract has also been reported [50]. They also investigated the antioxidant, antialgal and antifungal potential of green synthesized AgNPs. Similarly, another study reported the degradation of orange and blue dyes using AgNPs synthesized from *Adhatoda vasica* leaf extract [51]. Antibacterial activity of these nanoparticles has been studied against *Pseudomonas aeruginosa* MTCC 741 [52] and against gram-positive and gram-negative pathogenic bacterium [53]. Karthick et al. [54] and Bharathi et al. [55] described the green synthesis of AuNPs using *Adhatoda vasica Nees*, however, no application was discussed.

The aim of this work was to develop a simple, green, and cost-effective method to prepare AgNPs and study their physicochemical properties. In the present work we investigated the leaf extract of *Adhatoda vasica* which acted as the stabilizer and also as a reducing agent to prepare AgNPs. To the best of our knowledge, this is the first report for its application in the photocatalytic degradation of Amaranth, Allura red and Fast green dyes which are widely used in food, beverage, and cosmetic industries. One notable aspect of the work was its ability to achieve the rapid degradation of these dyes in just 60 min, showcasing remarkable efficiency. The synthesized AgNPs were characterized using different analytical tools such as UV Visible spectroscopy, Fourier Transformer Infrared (FTIR) Spectroscopy; High Resolution Transmission Electron Microscopy (HRTEM); X-ray Diffraction (XRD) analysis, Selected Area Electron diffraction (SAED) pattern, Scanning Electron Microscopy (SEM)and Energy Dispersive X-ray (EDX) and Thermogravimetric analyser (TGA). Furthermore, the degradation kinetics of these dyes is also presented.

## Materials and method

### Plant and chemicals

The leaves of *Adhatoda vasica* were obtained from Ahmedabad district, Gujarat, India (N 23°2′18.3768; E72°32′34.8468) during the summer season of 2022. The sample leaves were identified and authenticated by the Department of Botany, Gujarat University, Gujarat, India. The experimental research carried out using this plant, including the collection of plant material, complied with the Gujarat University guidelines and legislation. Silver nitrate (AgNO_3_) was purchased from Finar Limited, Gujarat. Allura red (E129), Amaranth (E123) and Fast green (E143) dyes were procured from Sigma Aldrich (Germany). Further, all other chemicals used were of analytical grade.

### Preparation of aqueous leaf extract of *Adhatoda vasica*

The fresh and undamaged leaves of *Adhatoda vasica* were first cleaned thoroughly using tap water and then washed carefully with Milli Q water. Thereafter the leaves were shade dried and 15 g of these dried leaves were chopped into small pieces and placed in a glass beaker. To this, 100 mL of Milli Q water was added, and the mixture was heated at 65 °C for 45 min. Subsequently, the solution was filtered using a Whatman filter paper no. 41, which had a pore size of 20–25 µm. The resulting leaf extract had a pale-yellow color and was used to reduce, cap, and stabilize the AgNPs. Finally, the extract was stored in a refrigerator at a temperature of 2 °C away from direct light and to prevent any agglomeration.

### Preparation of AgNPs using *Adhatoda vasica* leaf extract

A solution of 0.05 M AgNO_3_ was prepared using Milli Q water and 50 mL portion of the solution was taken in a conical flask. Since AgNO_3_ is sensitive to light and can convert into silver oxide in the presence of sunlight or bright light, the conical flask containing the AgNO_3_ solution was covered with aluminum foil. Next, the leaf extract was added dropwise to the AgNO_3_ solution under constant stirring at 480 rpm on a magnetic stirrer. After adding 7 mL of the extract, the color of AgNO_3_ solution changed from colorless to brown, indicating the formation of AgNPs. To purify the solution containing the green-synthesized AgNPs, it was centrifuged at 6000 rpm for 20 min until a clear supernatant was obtained. The supernatant was removed, and the resulting pellet was washed thrice with Milli Q water. The pellet was then dried overnight at room temperature and used for characterization and further analysis.

### Characterization of AgNPs

The prepared AgNPs were characterized by UV–visible spectrometer (JASCO V-630) in range of 200–800 nm. The absorption data was also used to find the band gap of AgNPs. To identify the functional groups, present in leaf extract, which are responsible for reducing, capping, and stabilizing action, FTIR (Agilent Micro lab) spectral analysis was performed. Using the powder form of AgNPs the FTIR spectrum was recorded from 500 to 4000 cm^−1^. XRD (Rigaku Smart Lab 2) was used to study the phase and crystalline structure of AgNPs. The XRD instrument was utilized with a 40 kV voltage and a 45 mA electric current, using Cu K-alpha radiation as the X-ray source. The diffractogram was recorded between 20 and 80 (2θ) angles. The Debye Scherrer formula was employed to determine the mean crystallite size of AgNPs, while qualitative and quantitative analysis of the elemental composition of AgNPs synthesized through the green method was conducted using EDX (Carl Zeiss Supra 55). Information regarding size, shape and distribution of particles were carried out using HRTEM (JEM-2100 high-resolution analytical TEM), operated at a voltage of 200 kV. TGA was employed to investigate the decomposition behaviour and thermal properties of AgNPs. The thermal analysis was conducted using a NETZSCH STA 2500 instrument in a nitrogen atmosphere, with temperature ranging from 28 to 1050 °C and a heating rate of 10 °C per min.

### Photocatalytic degradation of dye

The study aimed to investigate the photocatalytic degradation of Amaranth, Allura red, and Fast green dyes under sunlight using AgNPs synthesized through a green method. To prepare stock dye solutions, a concentration of 1 × 10^–3^ M was achieved by dissolving each dye in Milli-Q water. Four beakers were filled with 100 mL of the stock dye solution of Amaranth dye. In three of the beakers, 5 mg, 10 mg, and 15 mg of green synthesized AgNPs were added and thoroughly mixed. The fourth beaker served as a reference solution and did not have AgNPs. Subsequently, all four beakers were exposed to sunlight during the summer season, with an atmospheric temperature of 38 °C. The same procedure was repeated for Allura red and Fast green dyes. The UV–visible spectrophotometer was employed to measure the absorbance of all the dyes within the wavelength range of 300–800 nm. The maximum absorbance values were observed at 521 nm, 500 nm, and 624 nm for Amaranth, Allura red, and Fast green, respectively.

The percentage of dye degradation was found using the expression, % of degradation = 1-$\frac{At}{{A0}}$ × 100″, where $A_{0}$ is the initial absorbance of dye and $A_{t}$ is the absorbance of dye at time ‘*t*’. To study the kinetics of dye degradation reaction, the values of ln [C] vs t (1st order), 1/C vs t (2nd order), ln $\left( {\frac{C0}{{Ct}}} \right)$ vs t (Pseudo 1st order) were plotted and the coefficient of determination ($R^{2}$) for each graph was estimated to find the order of reaction.

## Results and discussion

### Process optimization for the synthesis of AgNPs using Adhatoda vasica

To prepare stable AgNPs, different parameters such as pH (3–9), volume of plant extract (5–15 mL), AgNO_3_ concentration (0.01–0.1 M), reaction temperature (25–45 °C) and reaction time (5–20 min) were studied. The optimum reaction conditions found were, pH 3; volume of extract, 7 mL; 50 mL, 0.05 M AgNO_3_; 25 °C temperature and 10 min of reaction time. The major constituents of *Adhatoda vasica* leaf extract includes pyrroloquinazoline alkaloids, vasicine, vasicol, adhatonine, vasicinone, vasicinol, vasicinolone, besides different glycosides, saponins, flavonoids and other phenolic compounds [54, 55]. The phytochemicals mainly responsible for the reduction of silver ions to AgNPs in solution are the phenols and alkaloids. The chemical reactions that are involved in converting silver ions into AgNPs are as follows,

1 $${\text{Ag}}\left( {{\text{NO}}_{{3}} } \right)\,\,\left( {{\text{colourless}}} \right)\, + \,{\text{Aq}}.\,{\text{plant}}\,{\text{extract}}\,\,\left( {{\text{pale}}\,\,{\text{yellow}}\,{\text{colour}}} \right) \to {\text{Ag}}^{ + } \, + \,{\text{NO}}_{{3}}^{ - }$$

2 $${\text{Ag}}^{ + } \, + \,{\text{e}}_{{{\text{aq}}}}^{ - } \left( {{\text{from}}\,\,{\text{phytochemicals}},\,\,{\text{phenols}},\,\,{\text{alkaloids}}} \right)\, \to \,{\text{Ag}}^{{\text{o}}} \,\left( {{\text{brown}}\,\,{\text{colour}}} \right)$$

The hydrated electrons (e_aq_^−^) act a strong reducing agent and thus reduce Ag^+^ ions into zero-valent Ag atoms (Ag^o^) (Eq. 2). The solution containing Ag^o^ was centrifuged and after decantation of the supernatant, AgNPs pellet was obtained. Additionally, flavonoids, tannins, and saponins serve as natural surfactants and capping agents in the formation of nanoparticles and prevent agglomeration [45, 46].

### UV–Visible spectroscopy

**Fig. 1:**
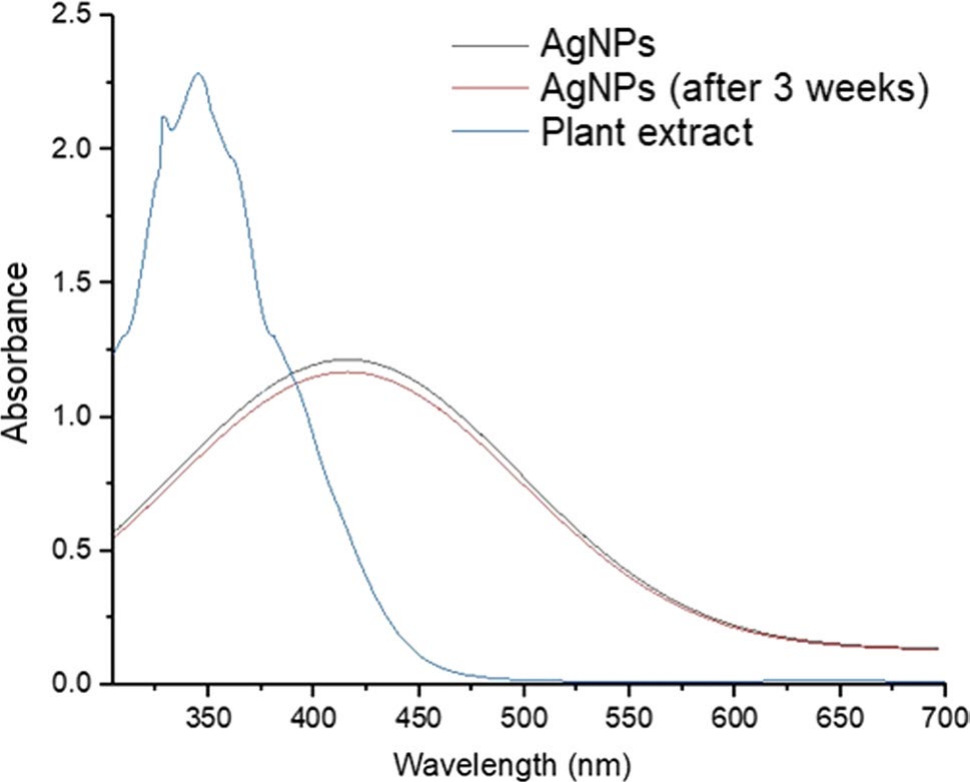
UV–visible spectra of AgNPs and the *Adhatoda Vasica* leaf extract

**Fig. 2:**
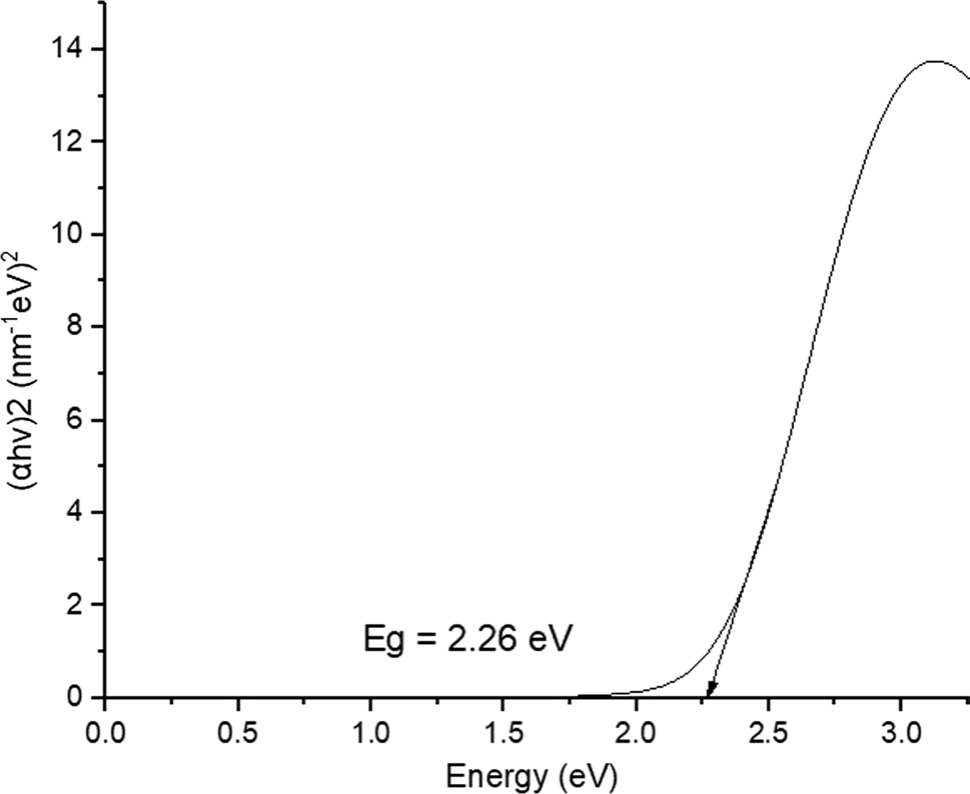
Tauc plot for optical band gap of AgNPs

**Fig. 3:**
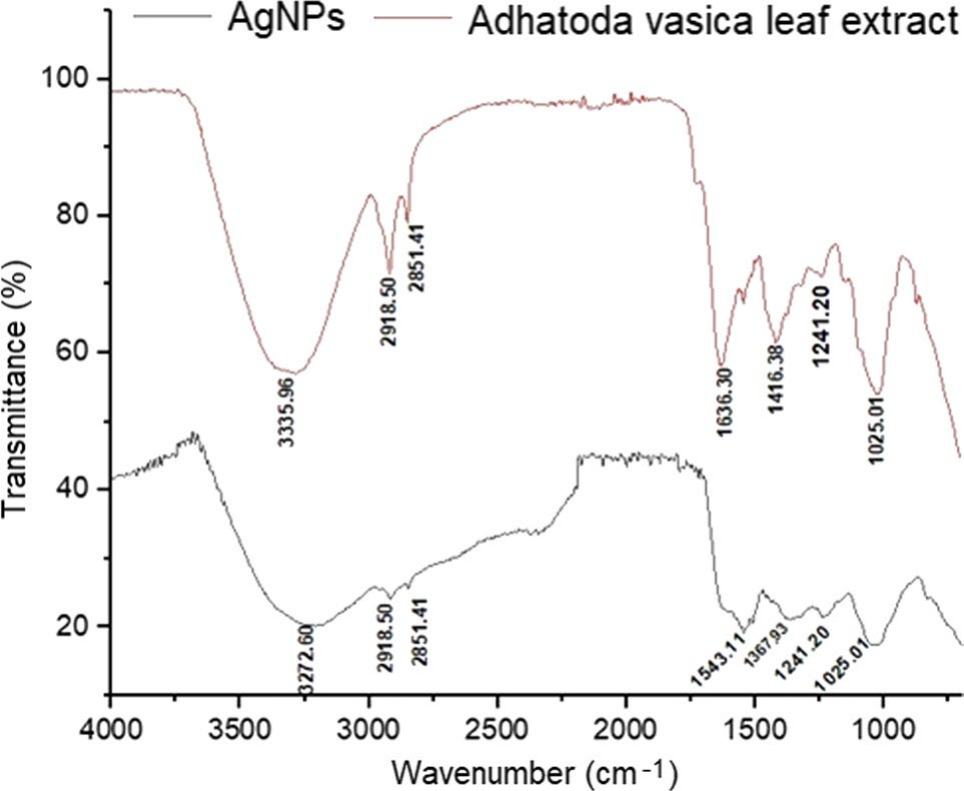
Comparison of FTIR spectra of *Adhatoda vasica* leaf and AgNPs

The prepared AgNPs showed an absorption peak at 419 nm (Fig. 1), which in accordance with a previous report, where AgNPs exhibits maximum absorption between 410 and 430 nm [56]. The leaf extract had an absorption maximum at 345 nm. The colour of green synthesized AgNPs remained stable for 3 weeks at 2 °C with no indication of oxidation, aggregation, and precipitation. This test was carried out in triplicate. However, there was a slight decrease in the absorbance with no change in the wavelength maxima, $\lambda_{max}$ after 3 weeks as shown in Fig. 3. This suggests that green synthesized AgNPs had high stability due to the presence of stabilizing and capping agents in the leaf extract. The UV–visible spectra were further used to find the optical band gap of AgNPs using Tauc plot, which is expressed as (αhν)^2^ = (hν-Eg), where α represents the absorptivity co-efficient and hν is the optical energy of the system [57]. The plot of (αhν)^2^ against hν and a tangent of curve extrapolated on X-axis gave the value of band gap (Eg) of AgNPs, as shown in Fig. 2. The Eg value of AgNPs obtained was 2.26 eV. Nanoparticles with a smaller Eg value are highly beneficial in various applications. Additionally, this characteristic allows for easier generation of electrons and holes, making these AgNPs more receptive to low-energy electromagnetic radiation, resulting in more efficient photocatalytic dye degradation [56].

### Fourier-transform infrared spectroscopy

The prepared AgNPs were analysed by FTIR spectroscopy to identify functional groups present in the biomolecules that assisted in the reduction, capping and stabilization of the NPs. These functional groups are responsible for bioreduction of silver ion (Ag^+^) into zero valent silver atom (Ag^0^). Figure 3 shows the FTIR spectra of *Adhatoda vasica* leaf extract and the synthesized AgNPs. The major vibrational peaks observed in leaf extract were, 3335.76 cm^−1^ due to stretching vibration of –OH (hydroxyl group), 2918.50 cm^−1^ due to C-H stretch (aliphatic), 2851.41 cm^−1^ on account of stretching vibration of –NH (amine group), 1636.30 cm^−1^ due to stretching vibration of C=O(carbonyl group), 1416.38 cm^−1^ can be ascribed to C=C bond (aromatic) stretching, 1241.20 cm^−1^ can be related to stretching vibration of –C–O– (aromatic ester) and 1025.01 due to stretching vibration of –COO^–^ (anhydride group). The O–H stretching vibration can be attributed to the O–H bond of flavonoids, saponins, and tannins. On the other hand, the C=C and C–H stretching vibrations indicate the presence of alkaloids, saponins, flavonoids, and tannins in *Adhatoda vasica* [45, 46]. According to the FTIR spectrum of the green synthesized AgNPs the peaks observed at 3272.60 cm^−1^, 2918.50 cm^−1^, 2851.41 cm^−1^, 1543.11 cm^−1^, 1367.93 cm^−1^, 1241.20 cm^−1^, and 1025.01 cm^−1^, were identical to the peaks that appeared in the FTIR spectrum of the leaf extract but the peak intensity was comparatively less compared to the leaf extract. However, some of the peaks of *Adhatoda vasica* leaf extract appeared to have shifted in the synthesized AgNPs, as follows, 3335.76 cm^−1^ to 3272.60 cm^−1^, 1636.30 cm^−1^ to 1543.11 cm^−1^ and 1416.38 cm^−1^ to 1367.93 cm^−1^. The results obtained confirmed the presence of phytochemicals in *Adhatoda vasica* leaf extract, which could have acted as reducing and capping agents for AgNPs.

### XRD analysis

**Fig. 4:**
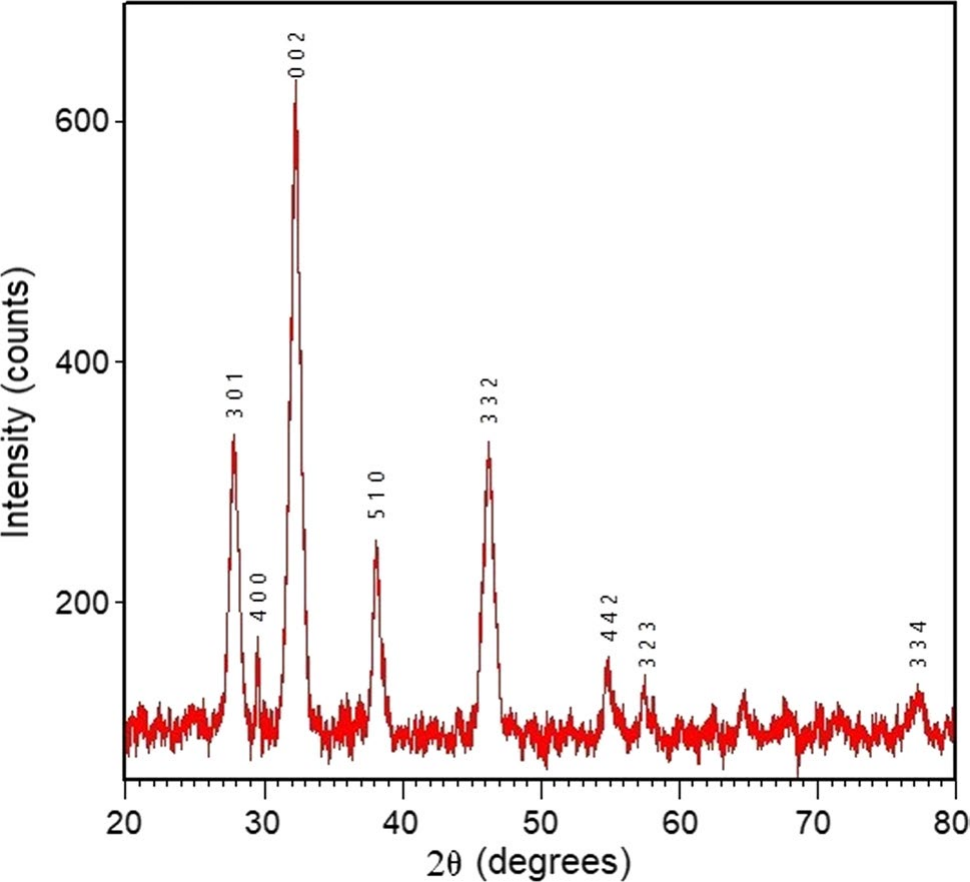
X-ray diffraction spectra of AgNPs

XRD analysis was employed to ascertain the crystalline composition of AgNPs. XRD analysis was used to identify the crystalline phase of AgNPs. The XRD spectrum of synthesized AgNPs is shown in Fig. 4. The presence of significant and acute diffraction peaks at 27.77°, 29.51°, 32.24°, 38.11°, 46.25°, 54.88°, 57.62°, and 77.21^o^related to (3 0 1), (4 0 0), (0 0 2), (5 1 0), (3 3 2), (4 4 2), (3 2 3), and (3 3 4) planes, respectively, suggests crystalline face centered cubic (FCC) phase [58]. The diffraction patterns of Ag can be correlated with the reference data found in the Joint Committee on Powder Diffraction Standards (JCPDS) files, specifically 96–900–8640 and 96–901-1667. Thus, from the observed pattern we can conclude face centered cubic structure of AgNPs (a = b = c = 3.9165 and α = β = ϒ 90°) [59].

Particle size plays a crucial role in the green synthesis of nanoparticles, and Scherer's formula is employed to calculate nanoparticle size using the full width at half maximum (FWHM) position [60].

$$\tau = \frac{k\lambda }{{\beta_{2\theta } \cos \theta }}$$

where τ signifies the crystal thickness, measured perpendicular to the reflecting plane, k is the Scherrer’s constant (for spherical particles the value is 0.9), wavelength (λ) of the X-ray radiation is 1.5405 Å, β_2θ_ (in radians) represents the width at half the maximum intensity, and θ is the Bragg’s angle [58]. The calculated crystallite range varied between 8 and 18 nm depending on the type of reflection used for the AgNPs. The average crystalline size of prepared AgNPs was 10.84 nm, which agrees with HRTEM result. The calculated data of lattice strain and dislocation density were found from the data using customized Scherrer formula as shown in Table 1 [61].

**Table 1:** XRD data of AgNPs prepared using leaf extract of Adhatoda vasica

2θ^o^	d-spacing (Å)	Crystalline size D (nm)	Dislocation density δ (× 10^14^) (Lin m^2^)	Strain ε (× 10^–4^) (Lin^−2^ m^−4^)
27.768	3.2102	8.0980	0.01525	1.8628
29.511	3.0244	12.8589	0.00605	1.0142
32.240	2.7744	18.2959	0.00299	0.6066
38.109	2.3595	13.4312	0.00554	0.6868
46.249	1.9614	8.1179	0.01517	0.9686
54.884	1.6715	8.2183	0.01481	0.9163
57.623	1.5984	8.5278	0.01375	0.7939
77.212	1.2345	9.2119	0.01178	0.6309

### HRTEM, EDAX and SEM analysis

**Fig. 5:**
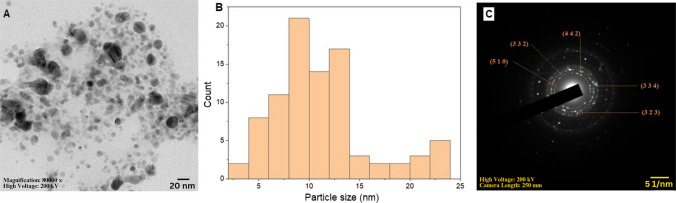
**a** HRTEM image, **b** particle size distribution and **c** selected area diffraction electron (SAED) pattern of AgNPs

The particle size and morphology of the synthesized AgNPs were determined by HRTEM analysis. According to the HRTEM micrograph (Fig. 5a), the size of AgNPs ranged between 3.88 and 23.97 nm and average particles size was 11.36 nm. The particles were spherical in shape and their size distribution is represented by a histogram (Fig. 5b). SAED pattern analysis gave an idea regarding crystalline nature of the prepared AgNPs. It showed diffraction rings with intermittent dots, suggesting crystalline nature of AgNPs (Fig. 5c). These concentric rings correspond to diffraction planes present in AgNPs and were indexed as (510), (332), (442), (323), and (334), which agrees with the XRD data.

**Fig. 6:**
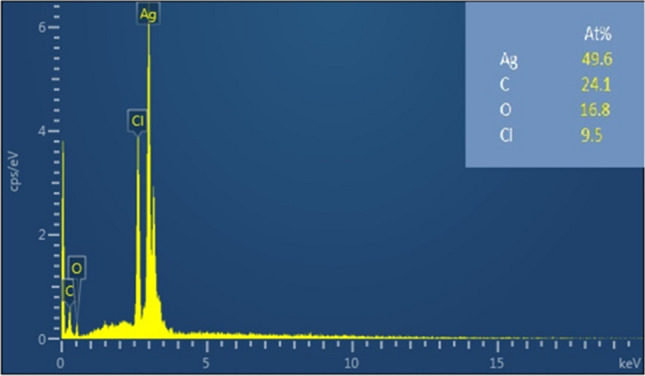
EDX pattern of AgNPs

**Fig. 7:**
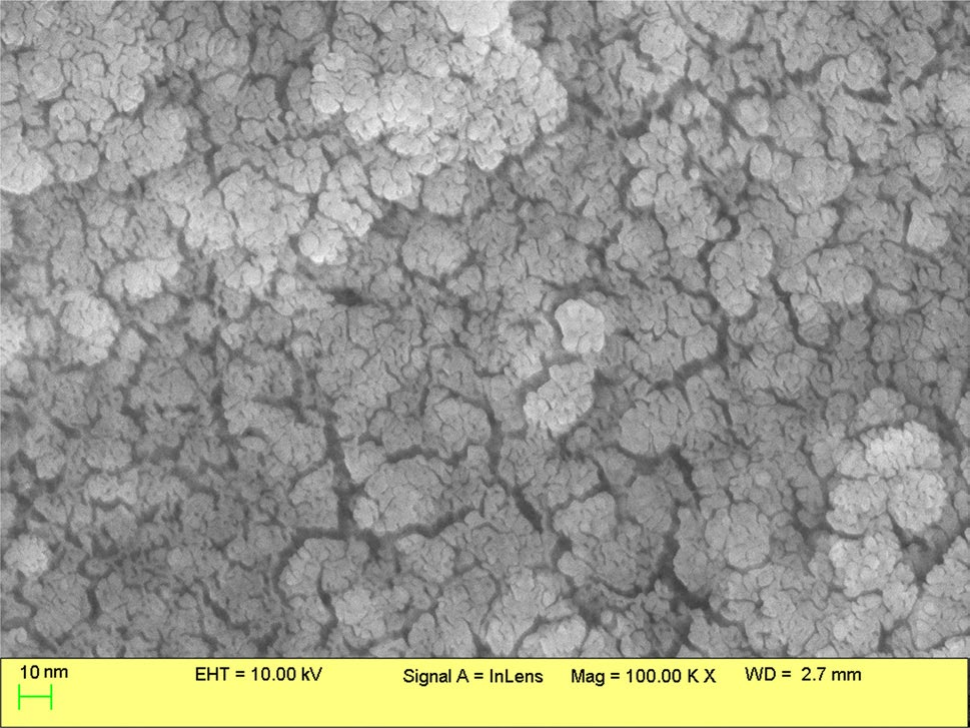
SEM micrograph of AgNPs

The elemental composition of green synthesized silver nanoparticles was assessed by EDX spectroscopy. A strong signal for silver [Ag (0), At. (49.6%)] was observed at 3 keV, which confirmed the presence of AgNPs (Fig. 6). The presence of carbon (At. 24.1%) and oxygen (At. 16.8%) in the EDX spectra can be attributed to the presence of capping and stabilizing agents present on the surface of AgNPs. The spectra also showed the presence of chlorine (At. 9%) as reported previously [55, 62]. The SEM micrograph showed spherical morphology of the AgNPs with the average size below 20 nm as presented in Fig. 7. The result further supports the size of particles estimated by XRD analysis.

### TGA analysis

**Fig. 8:**
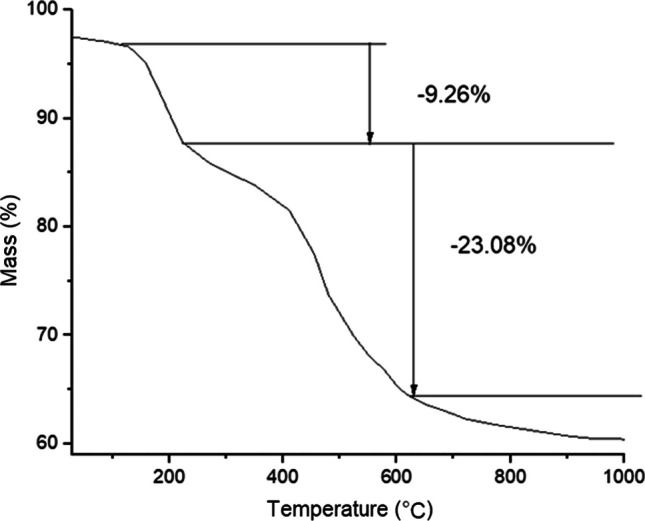
Thermogravimetric analysis of AgNPs

Thermal stability of green synthesized AgNPs and mass loss of AgNPs were determined by TGA analysis. TGA spectra shown in Fig. 8 displayed first mass loss of 9.26% between 96 and 220 °C due to the removal of water molecules present on the surface of AgNPs. The second mass loss (23.08%) observed up to 625 °C, was mainly due to the evaporation and decomposition of different phytochemicals present on surface of AgNPs, which serve as a capping and stabilizing agent in the green synthesis. Between 900 and 1000 °C, mass percentage becomes constant, around 60%. This shows that further mass loss was not observed due to the presence of silver metal.

### Photocatalytic degradation of dye

**Fig. 9:**
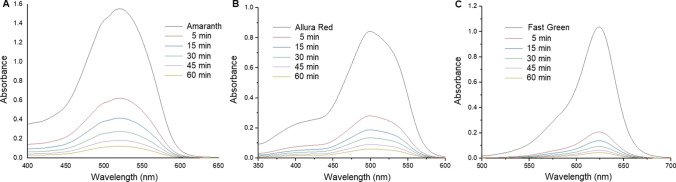
UV–visible spectra of degradation of (**a**) amaranth dye (**b**) allura red and (**c**) fast green dye using 5, 10, 15 mg of AgNPs. The absorbance measurements were made after tenfold dilution of the samples

To assess the photocatalytic activity of the prepared AgNPs, three different dyes namely Amaranth, Allura red and Fast green were studied. Initially, different amounts of AgNPs (5.0, 10.0 and 15.0 mg) were evaluated to optimize the best conditions for degradation. However, it was found that there was no significant difference in the percentage of dye degradation (89–92%). Thus, photodegradation was carried out by adding 5.0 mg AgNPs to 100 mL, 1.0 × 10^–3^ M aqueous solution of each dye in the presence of sunlight. It was observed that there was an immediate decrease in the absorption for all the dyes. With increase in the exposure time there was a corresponding decrease in the absorption peaks for the dyes. The UV–visible spectra in Fig. 9a–c shows time-dependent degradation of these dyes using 5.0 mg AgNPs. The absorbance measurements were made after tenfold dilution of the samples. The absorption peaks at 521 nm, 500 nm and 624 nm corresponding to Amaranth, Allura red and Fast green, respectively degraded substantially after 1 h of exposure. The results for experiment carried out using different amounts of AgNPs in the presence of sunlight is shown in Table 2. Significant degradation (in the range of 92–95%) was observed for the dyes using 5.0 mg AgNPs after 1 h exposure, however, the corresponding increase was not substantially higher with 10.0 mg or 15.0 mg AgNPs. To justify the photocatalytic activity of the prepared AgNPs, the experiment was also performed in the absence of sunlight, the results of which are represented in Table 3. Further, a control experiment was performed by keeping the dye solutions under sunlight without AgNPs, however, there was negligible degradation of the dye samples. To compare the efficiency of the prepared AgNPs for the degradation of dyes, an assessment of the results obtained from reported studies [63–70] for photocatalytic degradation of Amaranth, Allura red and Fast green is shown in Table 4.

**Table 2:** Degradation of food dyes using different amounts of AgNPs in the presence of sunlight

Time (min)	Degradation (%)								
	5 mg AgNPs			10 mg AgNPs			15 mg AgNPs		
	Amaranth	Allura red	Fast green	Amaranth	Allura red	Fast green	Amaranth	Allura red	Fast green
5	60.2	66.7	80.0	75.1	77.8	86.7	90.1	88.9	93.3
15	73.3	77.8	86.7	83.3	85.1	91.1	93.4	92.6	95.6
30	82.2	84.0	91.1	88.9	90.1	94.1	95.8	95.1	97.3
45	88.2	89.3	93.9	92.6	93.4	95.9	97.1	96.3	98.1
60	92.1	92.8	95.7	95.4	95.0	97.0	97.7	96.9	98.6

**Table 3:** Degradation of food dyes using different amounts of AgNPs in the absence of sunlight

Time (min)	Degradation (%)								
	5 mg AgNPs			10 mg AgNPs			15 mg AgNPs		
	Amaranth	Allura red	Fast green	Amaranth	Allura red	Fast green	Amaranth	Allura red	Fast green
5	33.4	37.6	48.2	44.5	46.8	54.5	62.3	59.5	63.0
15	42.1	44.2	55.7	46.2	52.1	59.2	65.4	64.6	65.8
30	57.2	58.1	63.2	58.4	60.2	68.3	66.7	66.3	67.4
45	59.8	60.2	65.6	60.3	63.5	69.8	68.1	67.2	70.2
60	63.2	64.0	67.3	65.4	67.5	72.5	70.2	69.5	73.4

**Table 4:** Comparison of previously reported methods with the present work for degradation of amaranth, allura red and fast green

Dye	Material/Method	Degradation (%)	Time	References
Amaranth	Magnetic Cobalt-embedded Carbon Nanofiber for activation of ozone and degradation	100%	40 min	[63]
	Sonoelectrochemical process	50%	90 min	[64]
	Zeolite Y encapsulated with Fe-TiO_2_ and ultrasound assisted	97.5%	120 min	[65]
	AgNPs from Adhatoda vasica	97.7%	60 min under sunlight	Present Study
Allura red	AgNPs from *Ekebergia capensis* plant extract in the presence of NaBH_4_	100%	45 min under visible light	[66]
	Polyaniline/TiO_2_ nanocomposite	70%	80 min under UV radiation	[67]
	Fe-Zn nanoparticles	66%	120 min	[68]
	AgNPs from Adhatoda vasica	96.9%	60 min under sunlight	Present Study
Fast green	Oxidized graphite supported La_2_O_3_/ZrO_2_ nanocomposite	89%	90 min	[69]
	Chitosan membrane embedded with ZnO	57.9% under solar light and 71.45 under UV light irradiation	110 min for solar and UV irradiation	[70]
	Chitosan membrane embedded with ZnO/CuO	60.23% under solar light and 91.21 under UV light irradiation	110 min for solar and UV irradiation	[70]
	AgNPs from Adhatoda vasica	98.6%	60 min under sunlight	Present study

Based on the current research, it has been determined that to treat a typical wastewater containing 15 mmoles of dyes, the photocatalytic dye degradation requires 2.8 g of AgNPs using the present method. Additionally, this amount of AgNPs can be obtained from 15 g of leaves from the *Adhatoda vasica* plant. These nanoparticles possess the ability to degrade the dyes within 60 min with approximately 96% efficiency. However, future advancements in this field would aim to enhance the efficiency, reduce costs, and shorten the degradation time by utilizing AgNPs-based nanocomposites, such as those synthesized using environmentally friendly techniques with activated carbon, graphene oxide, etc. Furthermore, incorporating silica spheres to support the AgNPs could enhance the catalytic efficiency of nanoscale materials. Consequently, the resulting materials would exhibit greater versatility in degrading various types of dyes.

**Fig. 10:**
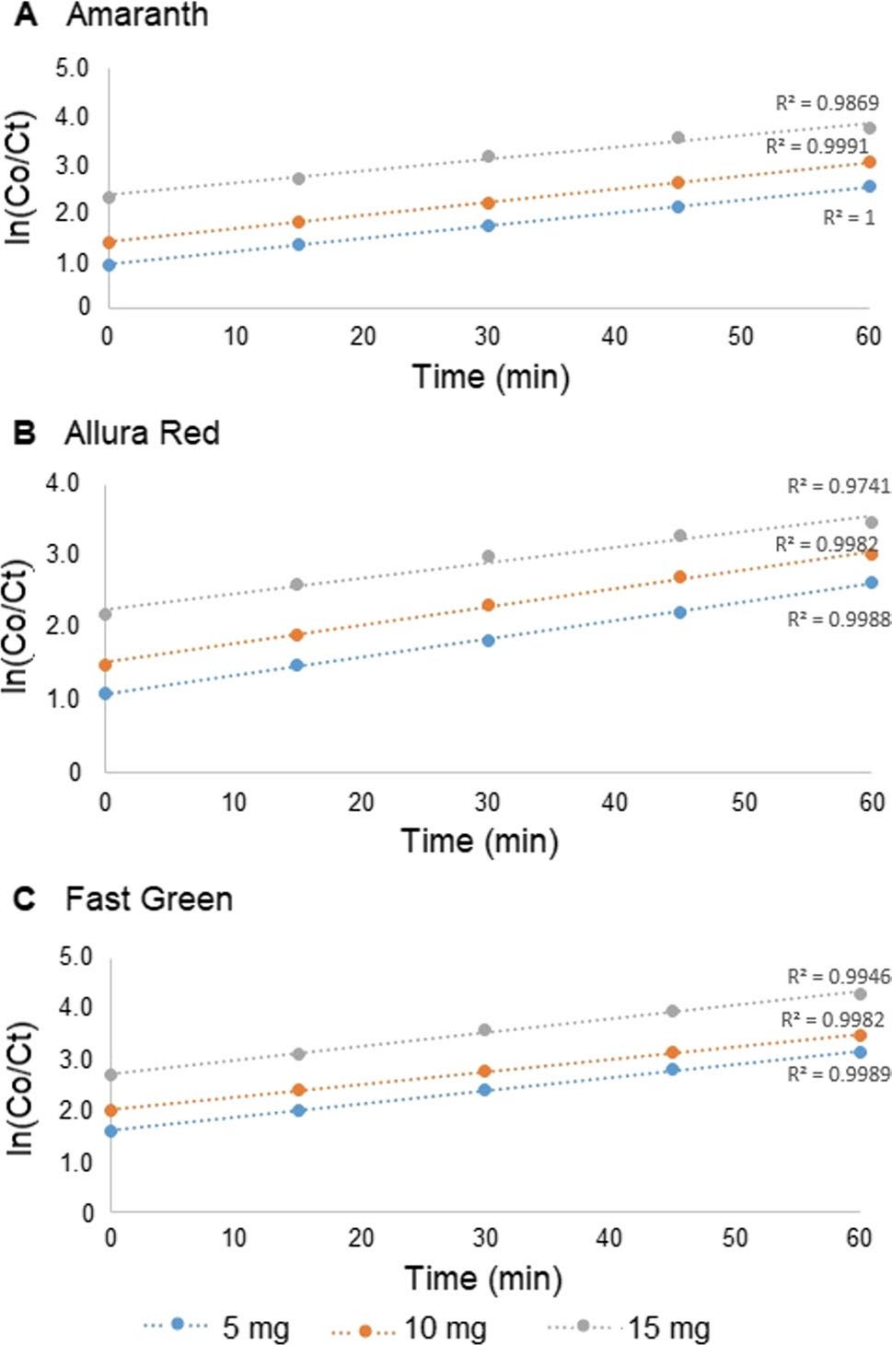
Plots of ln $\left( {\frac{C0}{{Ct}}} \right)$ versus t for (**a**) amaranth dye, (**b**) allura red and (**c**) fast green dye

Further, the kinetics of dye degradation showed pseudo-first order as evident from the plots of ln(C_0_/C_t_) vs. irradiation time ‘*t*’ for the dyes, where C_t_ and C_o_ represent the concentration of the dye at time ‘t’ and ‘0’, respectively (Fig. 10). The plots showed a linear relationship for all the dyes and the slope values corresponded to the pseudo-first order rate constant (*k*) for the degradation of the dyes. The value of *k* was found for Amaranth, Allura red and Fast green was 6.02 × 10^−2^, 6.19 × 10^−2^ and 8.43 × 10^−2^ min^−1^, respectively. These values of rate constant, *k* was essentially dependent on the chemical structure of the dyes. Thus, it can be concluded that the prepared AgNPs were highly efficient for the degradation of these dyes.

## Conclusion

This study presents a promising approach to produce AgNPs using leaf extract of *Adhatoda Vasica*. The developed method eliminates the need for organic solvents, surfactants, capping agents, and templates. It highlights the dual functional ability of the *Adhatoda Vasica* extract solution. As a result, this fabrication method is straightforward, environmentally friendly, cost-effective, and sustainable. The synthesized AgNPs showed high efficiency to degrade three dyes namely, Amaranth, Allura Red and Fast green within 1 h. Further, it was possible to obtain 2.8 g of AgNPs using 15 g of *Adhatoda vasica* leaves which could efficiently degrade 15 mmoles of dyes present in water samples. This efficacy showcases their potential as efficient photocatalysts for industrial effluent treatment. Therefore, this technique holds significant promise for the fabrication of AgNPs and their application in environmental remediation.

## Data Availability

There is no data to report other than that presented in this study.
